# The impact of COVID-19 pandemic on emotional and behavioral problems of children with autism spectrum disorder and developmental delay aged 1–6 years in China

**DOI:** 10.3389/fpsyt.2023.1134396

**Published:** 2023-02-24

**Authors:** Yanan Zhao, Yanan Luo, Rong Zhang, Xiaoying Zheng

**Affiliations:** ^1^School of Population Medicine and Public Health, Chinese Academy of Medical Sciences/Peking Union Medical College, Beijing, China; ^2^China Center for Health Development Studies, Peking University, Beijing, China; ^3^Asia-Pacific Economic Cooperation Health Sciences Academy, Peking University, Beijing, China; ^4^Department of Global Health, School of Public Health, Peking University, Beijing, China; ^5^Neuroscience Research Institute, Peking University, Beijing, China; ^6^Key Laboratory for Neuroscience of the Ministry of Education, Key Laboratory for Neuroscience of the Ministry of National Health Commission, Department of Neurobiology, School of Basic Medical Sciences, Peking University Health Science Center, Beijing, China; ^7^Autism Research Centre, Peking University Health Science Centre, Beijing, China

**Keywords:** COVID-19, Child and Adolescent Psychiatry, development delay, autism spectrum disorder, emotional and social development

## Abstract

**Introduction:**

The COVID-19 pandemic outbreak have caused increased levels of emotional and behavioral problems, particularly among people with pre-existing mental health conditions. Young individuals with autism spectrum disorders (ASD) and developmental delay (DD) are particularly at risk due to their vulnerability. The purpose of this study was to look into the different effects of the COVID-19 pandemic on 1–6-year-old children with ASD and DD.

**Methods:**

Parents and guardians of children with ASD completed an online survey that included questions about their children’s socio-demographics characteristics, the effects of the COVID-19 outbreak on their health, and what they needed in order to deal with the conditions of the pandemic.

**Results:**

This study compared 4,138 children with ASD to 711 children with DD. Children with ASD had a higher risk of having more emotional and behavioral problems than children with DD (OR 1.38, 95% CI 1.12–1.70). Compared to parent-oriented rehabilitation at home, discontinuing rehabilitation had a higher likelihood of negative emotional and behavioral change (OR 1.67, 95% CI 1.41–1.98). Having teachers’ online support had a higher likelihood of negative emotional and behavioral change for ASD children (OR 1.26, 95% CI 1.03–1.54).

**Conclusions:**

This article provided evidence that children with developmental disabilities, particularly ASD, were at risk for a variety of challenges to their emotional functioning during the COVID-19 period, and that online support was not an ideal way for children with ASD to receive effective educational intervention in China.

## Introduction

The COVID-19 pandemic is a global health crisis that has resulted in a public health emergency. It has caused a high level of psychological stress in the general population (1) as well as an increased risk of emotional and behavioral problems in people who already have a mental health condition (2).

The massive effects of the COVID-19 pandemic have forced both the formal and informal sectors to halt operations, including the educational sector (3). Individuals with special needs are receiving fewer, if any, crucial therapy hours (e.g., speech therapy, behavioral therapy) and classroom time than they would normally (4), particularly children. Many parents have considered home education as an alternative mode of intervention during the pandemic (5). Several studies have confirmed that home education was an additional useful learning method for children during the COVID lockdown (3, 5, 6). However, implementing home education presents numerous challenges (7), and the provision of online educational intervention is insufficient (3, 8). The need for novel approaches and the continuity of care for those with chronic health problems during the pandemic cannot be overstated (9).

Among vulnerable populations, children with severe developmental disabilities are of particular concern regarding how the COVID-19 outbreak may have impacted their wellbeing and health (7, 8). Two common developmental disorders are autism spectrum disorders (ASD) and developmental delay (DD). ASD is a range of neurodevelopmental disorders that are characterized by impairments in social interaction and communication and restricted, repetitive behaviors (10). A child with developmental delay (DD) has delays in language, fine and gross motor functioning, sensory integration, cognitive functioning, or communication, as well as behavioral and socio-psychological problems during infancy or early childhood (11, 12). The COVID-19 outbreak has undoubtedly resulted in a rapidly shifting social situation, which may exacerbate the difficulties of children with developmental disabilities (13), particularly children with ASD. Because they are vulnerable to the effects of prolonged isolation or quarantine, they may struggle to adapt to this new norm, especially given the disorder’s inflexibility and insistence on sameness (10). Furthermore, children with ASD are more likely to have comorbidities such as anxiety and learning disabilities, which present additional challenges to be dealt with during the COVID-19 pandemic (4, 7, 14, 15). We may discover that people with developmental disabilities are more vulnerable in general, and that disruptions in routine caused by COVID-19 can cause major emotional and behavioral upheaval (16); however, this has not been thoroughly researched in potentially higher-risk groups, such as children with ASD and DD. We don’t know how the effects differ between severe developmental disorders. Children with ASD may have expressive communication challenges, making it difficult to communicate pain (17, 18), while children with DD may have delays in development of language and intellectual abilities. We can better study the significance of online educational intervention and gain a better understanding of different neurodevelopmental conditions by comparing the performance of the two types of children during the epidemic.

The government in China has taken strict measures to control the pandemic in order to protect vulnerable people from COVID-19 while not overburdening the health service. From February 2020, most Chinese residents were required to stay at home and maintain social distance. Although some institutions reopened after April, the primary focus remained on online education. Most institutions or schools reopened after June, but the hours of operation were not consistent due to the epidemic. Suspension of therapy courses for children and adolescents with specific requirement may result in missed opportunities for basic skill development (4, 13, 15–19). Worse, staying at home puts children with special educational needs and their families in an unusually stressful situation (20). According to recent surveys in China, the prevalence of ASD was 0.7% (21), while the prevalence of DD was 4.5% (22). As a developing country with a large population and an ineffective social security system, China faces a health crisis.

Beginning with the negative emotional and behavioral effects of the COVID-19 outbreak on children with ASD and DD, the overall goal of this study is to investigate child statute changes in Chinese families with children with ASD and DD during lockdown. We aimed to (1) investigate the different effects of the COVID-19 pandemic on the emotional and behavioral change of children with ASD and DD; (2) investigate whether any pre-pandemic sociodemographic or child characteristics would predict a negative impact of the pandemic on children with ASD and DD; and (3) investigate the effects of home education and family needs in the COVID-19 lockdown. Based on previous evidence (23), emotional and behavioral problems during the pandemic were hypothesized to predict a poor outcome.

## Materials and methods

### Data source

This study used data from the Survey on Family Circumstances and Demand for Support and Resources among Autistic Children in China (FCDSR). It was a survey that was distributed to members of the AlsoLife online patient community. The Quality Assurance staff at the China Association of Rehabilitation of Disabled Persons (CARDP) reviewed the survey for editorial and technical suggestions, which aimed to describe the family information, treatment, rehabilitation subsidies, and health expenditure of children with ASD. The other details of the survey have been described elsewhere (24). We did not use a sampling design because there is no nationwide ASD survey in China. A pilot field study (*N* = 20) was conducted to refine the instrument and data collection procedures, and the results indicated that respondents generally understood the questionnaire, so only minor wording changes were made.

**FIGURE 1:**
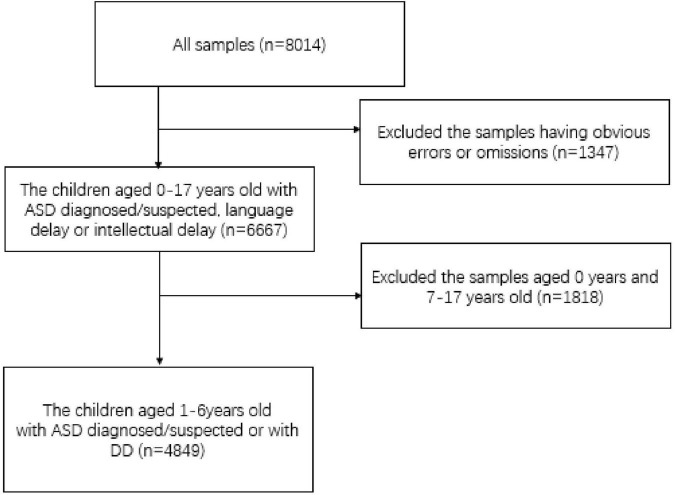
Flow chart.

The online survey was completed by parents and guardians of people with ASD or DD, of which 78.1% were mothers, 19.7% were fathers, and 2.2% were other guardians. Additionally, ASD advocacy and family support networks were used to distribute and directly encourage survey participation. During the questionnaire collection and data cleaning process, data with obvious errors or omissions were removed. A total of 8,014 households were analyzed, with 4,849 households included in this study, in which 4,138 households had children with ASD and 711 households had children with DD. Families with children diagnosed or suspected of having ASD or DD were invited to participate if their children met the following criteria: (1) age 1–6 years and diagnosed or suspected of having ASD through a hospital diagnosis; (2) diagnosing hospital and diagnosing hospital department both having diagnostic qualifications. Exclusion criteria were patients with physical disabilities, cerebral palsy, or epilepsy as comorbidities. The selection procedure is depicted in Figure 1. Thirty-one provinces and a total of 385 cities in China were included. Given the lack of a large-scale survey of children with ASD or DD in China, the second national sample survey of disabled people in China could also be used as a reference. These samples are representative (see Supplementary Tables 1, 2).

### Measures

The survey included questions about the impact of the COVID-19 outbreak on their wellbeing as well as the requirements for dealing with the pandemic. Each multiple-choice question allowed participants to choose only one item. This measure of emotional and behavioral change was reported by parents who were asked about their children’s emotional and behavioral changes (e.g., emotional reactions to stress, emotional self-regulation system’s stability and emotional and behavioral problems related to ASD or DD) during the COVID-19 pandemic lockdown. Specifically, a scale of 1 = “improved,” 2 = “no change,” and 3 = “worse” was used. Demographic variables, family socioeconomic variables, and family treatment history variables were used as control variables in this study. The demographic variables included the age of the children, their gender, and the number of children in the household, and having comorbidities or not. The age was the age at the time of the survey. The comorbidities referred to neurodevelopment disorders, including intellectual disabilities (ID) and attention deficit and hyperactivity disorder (ADHD) in this study. Information on family sociodemographic and medical history was gathered. The income of families was divided into three categories: below average, average, and above average. According to the data distribution, the below average group had an annual income of less than $12,327 (RMB80,000), the average group had an annual income between $12,327 (RMB80,001) and $23,112 (RMB150,000), and the above average group had an annual income greater than $23,112 (RMB150,000).

### Statistical analysis

The final raw data were downloaded from WJX Forms into a Microsoft Excel file for analysis using SPSS software. Descriptive statistics were used to provide baseline information concerning survey participants’ children with ASD. Logistic regressions were performed to investigate whether the socio-demographic or clinical characteristics of any individuals with ASD would predict a greater frequency and intensity of emotional and behavioral problems following the COVID-19 outbreak. Associations between predictors and independent variables were reported by odds ratios (ORs) and their 95% confidence intervals (CIs). All the estimated costs were converted to US dollar (US$) values in January 2021, when one US$ was equivalent to about 6.49 Chinese yuan. All statistical analyses were conducted using SPSS 22.0 for Windows (SPSS Inc., Chicago, IL, USA).

### Consent and ethics approval

All families provided electronic informed consent before enrollment. We assert that all procedures contributing to this work comply with the ethical standards of the relevant national and institutional committees on human experimentation. All procedures involving human subjects/patients were approved by the ethics committee of Peking University Institutional Review Board and approval number is IRB00001052-20016.

## Results

### Study population

A total of 4,849 eligible children (78.8%) were recruited for the study. Table 1 presents characteristics of the participating children. Compared with children with DD, children with ASD had a greater proportion of parents with higher education (father with a higher education degree, 66.92% vs. 59.63%; mother with a higher education degree, 66.67% vs. 59.35%; both, *p* < 0.01). Compared with children with DD, children with ASD had a smaller proportion of having comorbidities (19.33% vs. 24.47%, *p* < 0.01). Age, gender, and family income did not differ significantly between the two groups.

**TABLE 1:** Characteristics of ASD and DD groups.

	ASD (*N* = 4,138)		DD (N = 711)		*P*-value
	**Mean/Number**	**SD/%**	**Mean/Number**	**SD/%**	
Age (years)	4.21	1.23	4.21	1.26	0.130
**Gender of child**					
Male	3,451	83.40	542	76.23	<0.001
Female	687	16.60	169	23.77	
**Only children**					
No	1,969	47.58	355	49.93	0.255
Yes	2,169	52.42	356	50.07	
**Comorbidity^a^**					
No	3,338	80.67	537	75.53	0.002
Yes	800	19.33	174	24.47	
**Higher education degree-father**					
No	1,369	33.08	287	40.37	<0.001
Yes	2,769	66.92	424	59.63	
**Higher education degree-mother**					
No	1,379	33.33	289	40.65	<0.001
Yes	2,759	66.67	422	59.35	
Family annual income^b^	22448.87	25124.34	19521.42	19295.19	0.067

ASD, autism spectrum disorder; DD, developmental delay. ^a^Including intellectual disabilities (ID) and attention deficit and hyperactivity disorder (ADHD). ^b^USD, $.

### Emotional and behavioral impact at the outbreak of the pandemic

Table 2 shows the impact of the pandemic outbreak on children and parents. For the ASD group and the DD group, most of the families engaged in parent-oriented educational intervention (59.35% vs. 54.57%), while more than 20% of the families ceased intervention in the lockdown (22.40% vs. 27.14%). Changes in behavioral or emotional problems had some differences between the two groups, where more children in the ASD group showed a decline than the DD group (22.31% vs. 18.00%).

**TABLE 2:** The changes of children and parents after the outbreak of COVID-19.

	ASD (*N* = 4,138)		DD (N = 711)	
	***N***	**%**	***N***	**%**
**Rehabilitation type**				
Parent oriented	2,456	59.35	388	54.57
Teachers’ online support	755	18.25	130	18.28
Cease	927	22.40	193	27.14
**Emotional and behavioral change**				
Improved	1,573	38.01	286	40.23
No change	1,642	39.68	297	41.77
Worse	923	22.31	128	18.00
**Time spent between parents and children**				
Shorter	195	4.71	18	2.53
No change	1,102	26.63	212	29.82
Longer	2,841	68.66	481	67.65
**Parental pressures change**				
More	2,577	62.28	430	60.48
No change	1,241	29.99	231	32.49
Less	320	7.73	50	7.03

ASD, autism spectrum disorder; DD, developmental delay.

Most families in both groups increased their parental time spent with their children. In both groups, parental pressures were greater than before.

### Predictors of negative impact on emotional and behavioral changes in the pandemic

The logistic regression model results showed that children with ASD had a higher likelihood of experiencing negative emotional and behavioral change (OR 1.38, 95% CI 1.12–1.70; Table 3). Compared with parent-oriented educational intervention at home, ceasing intervention had a higher likelihood of negative emotional and behavioral change (OR 1.67, 95% CI 1.41–1.98; Table 3). Children with comorbidities were associated with a more than one-fold likelihood of having negative emotional and behavioral change (OR 1.52; 95% CI 1.29–1.80; Table 3), and having conflicts with other family members was associated with a more than one-fold likelihood of having a poor mental status (OR 1.44; 95% CI 1.22–1.70; Table 3). Having a family income of average and above average was associated with a lower likelihood of negative emotional and behavioral change (OR 0.82; 95% CI 0.68–0.98 for average income families and OR 0.81; 95% CI 0.67–0.98 for above average income families; Table 3) compared to those having an income below average. Parental time spent with children had an effect on children’s emotions and behaviors. If the amount of time spent had not changed or had increased, the likelihood of having more negative emotional and behavioral changes decreased (OR 0.43; 95% CI 0.31–0.61 for families where there was no change in time spent with children; OR 0.41; 95% CI 0.30–0.57 for families where time spent with children increased; see Table 3).

**TABLE 3:** Logistic regression analysis for variables predicting the negative emotional and behavioral change* of children.

Characteristics	(*N* = 4,849)		
	**Odds ratio**	**95% CI**	
**Disorder**			
DD	1.00	reference	
ASD	1.38	1.12	1.70
**Rehabilitation type**			
Parent oriented	1.00	reference	
Teachers’ online support	1.19	0.99	1.44
Cease	1.67	1.41	1.98
**Rehabilitation hours**			
< 4 h a day	1.00	reference	
≥ 4 h a day	0.85	0.68	1.05
**Only children**			
No	1.00	reference	
Yes	0.96	0.83	1.11
**Gender of child**			
Male	1.00	reference	
Female	1.03	0.85	1.11
**Age of child**			
1–3 years	1.00	reference	
4–6 years	1.15	0.98	1.34
**Comorbidity^a^**			
No	1.00	reference	
Yes	1.52	1.29	1.80
**Household income**			
Below average	1.00	reference	
Around average	0.82	0.68	0.98
Above average	0.81	0.67	0.98
**Higher education degree-father**			
No	1.00	reference	
Yes	1.04	0.86	1.27
**Higher education degree-mother**			
No	1.00	reference	
Yes	1.11	0.91	1.34
**Time spent between parents and children**			
Shorter	1.00	reference	
No change	0.43	0.31	0.61
Longer	0.41	0.30	0.57

*Emotional and behavioral change is “worse” in the survey. ASD, autism spectrum disorder; DD, developmental delay. ^a^Including intellectual disabilities (ID) and attention deficit and hyperactivity disorder (ADHD).

Child gender, age, number of children in the household, and parent’s educational level were not significantly associated with negative emotional or behavioral change of children (Table 3).

**FIGURE 2:**
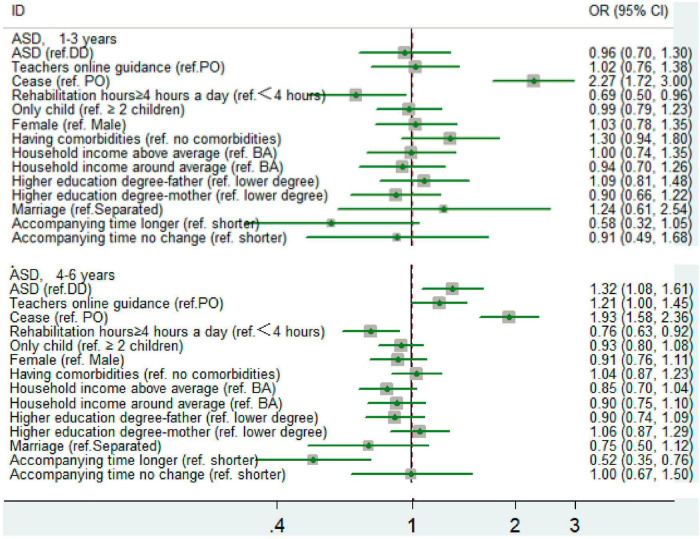
The odds of negative emotional and behavioral change in two group.

Stratifying by age groups resulted in significant differences (*p* < 0.05). For the 4–6-year-old group, the likelihood of negative emotional and behavioral change for children with ASD was higher than children with DD (*P* < 0.05). However, for the 1–3-year-old group, the difference between children with ASD and DD was not significant. For the 4–6-year-old group, the amount of time spent with parents had significant influence on the likelihood of negative emotional and behavioral change in two age groups. The longer the accompanying time the less likely the children had negative emotional and behavioral change (Figure 2).

Logistic regression was performed on children with ASD and DD to identify the factors that influenced each group. Table 4 shows the outcomes.

**TABLE 4:** Logistic regression analysis for variables predicting the negative emotional and behavioral change* of children in two groups.

Characteristics	ASD group (*n* = 4,138)			DD group (*n* = 711)		
	**Odds ratio**	**95% CI**		**Odds ratio**	**95% CI**	
**Rehabilitation type**						
Parent oriented	1.00	reference		1.00	reference	
Teachers’ online support	1.26	1.03	1.54	0.80	0.45	1.42
Cease	1.72	1.44	2.07	1.95	1.33	2.88
**Rehabilitation hours**						
< 4 h a day	1.00	reference		1.00	reference	
≥ 4 h a day	0.84	0.67	1.05	0.91	0.46	1.78
**Only children**						
No	1.00	reference		1.00	reference	
Yes	0.97	0.84	1.13	0.91	0.61	1.36
**Gender of child**						
Male	1.00	reference		1.00	reference	
Female	1.00	0.81	1.22	1.13	0.72	1.78
**Age of child**						
1–3 years	1.00	reference		1.00	reference	
4–6 years	1.15	0.97	1.35	1.11	0.72	1.71
**Comorbidity^a^**						
No	1.00	reference		1.00	reference	
Yes	1.59	1.33	1.90	1.28	0.82	2.00
**Household income**						
Below average	1.00	reference		1.00	reference	
Around average	0.84	0.69	1.02	0.74	0.45	1.22
Above average	0.83	0.68	1.01	0.72	0.43	1.22
**Higher education degree-father**						
No	1.00	reference		1.00	reference	
Yes	1.06	0.86	1.30	1.02	0.61	1.72
**Higher education degree-mother**						
No	1.00	reference		1.00	reference	
Yes	1.13	0.92	1.39	0.94	0.55	1.59
**Time spent between parents and children**						
Shorter	1.00	reference		1.00	reference	
No change	0.40	0.29	0.55	0.62	0.19	2.04
Longer	0.35	0.26	0.48	0.80	0.25	2.54

*Emotional and behavioral change is “worse” in the survey. ASD, autism spectrum disorder; DD, developmental delay. ^a^Including intellectual disabilities (ID) and attention deficit and hyperactivity disorder (ADHD).

For the ASD group, the logistic regression model results showed that compared with parent-oriented intervention at home, ceasing rehabilitation had a higher likelihood of negative emotional and behavioral change (OR 1.67, 95% CI 1.41–1.98; Table 4) and having teachers’ online support had a higher likelihood of negative emotional and behavioral change in children (OR 1.26, 95% CI 1.03–1.54; Table 4). Having children with comorbidities was associated with a more than one-fold likelihood of having negative emotional and behavioral change in children (OR 1.59; 95% CI 1.33–1.90; Table 4). If the accompanying time did not change or was longer than it previously had been, the likelihood of negative emotional and behavioral changes occurring was lower (OR 0.40; 95% CI 0.29–0.55 for families where time spent did not change and OR 0.35; 95% CI 0.26–0.48 for families where time spent was longer than previously; see Table 4). Child gender, age, number of children in the household, parents’ education, and household income were not significantly associated with negative emotional and behavioral change in children (Table 4).

For the DD group, however, only ceasing rehabilitation had a higher likelihood of negative emotional and behavioral change (OR 1.95, 95% CI 1.33–2.88; Table 4).

### What is needed to deal with conditions of the pandemic

A total of 4,849 survey participants responded to the open question about what could be done to help deal with the ongoing pandemic. The most commonly reported requirement was more professional one-to-one online support (53.93%), followed by financial support (28.05%), online knowledge training (16.33%), and others (1.69%); see Table 5.

**TABLE 5:** Responses to the open-ended question about what could be of help to deal with the conditions of the ongoing pandemic.

	ALL	ASD	DD
Financial support	1,360 (28.05)	1,134 (27.40)	226 (31.79)
Professional one-to-one online support	2,615 (53.93)	2,251 (54.40)	364 (51.20)
Online knowledge training	792 (16.33)	684 (16.53)	108 (15.19)
Others	82 (1.69)	69 (1.67)	13 (1.83)
Sum	4,849 (100.00)	4,138 (100.00)	711 (100.00)

ASD, autism spectrum disorder; DD, developmental delay.

In the ASD group, more professional one-to-one online support was needed by a higher percentage of parents compared to the DD group.

## Discussion

This was the first study in China to use a national survey to investigate COVID-19’s negative impact on children with ASD and DD. This study yielded several key findings.

First, we discovered that 22.31% of children with ASD exhibited increased emotional and behavioral issues during the lockdown. For the children with DD, 18% experienced increased emotional and behavioral problems. According to the theory of mind, the age of 4–5 years old is critical for the development of social abilities (25, 26). However, governments and policymakers all over the world, including China, chose school closure and home confinement as two necessary measures to limit the spread of COVID-19 infection, making it impossible for preschoolers with ASD and DD to improve their ability during their critical developmental period. Child isolation was detrimental to the emotional wellbeing of children with developmental disabilities (27, 28). Due to COVID-19, home schooling occurred involuntarily and was associated with feelings of loneliness, negatively impacting the children’s mental health and worsening pre-existing psychiatric disorders and behavioral problems (14, 23, 29). Our findings were slightly lower than other studies; for example, 45% of the sample had a worsening of their pre-existing psychiatric disorder (30), and 35.5% and 41.5% of children with ASD presented with more intense and frequent behavior problems, respectively (31). On the one hand, we used a larger sample, which may reduce the percentage of negative outcomes; on the other hand, differences between countries may cause some difference, as a Chinese small sample survey found that one-third of children’s social and emotional status had worsened (32). In our study, a recall bias could lead to underestimation.

Second, the COVID-19 pandemic affected children with ASD differently than children with DD. Children with ASD had a higher likelihood of negative emotional and behavioral changes than children with DD, particularly those aged 3–6 years. The abrupt removal of resources during the first national lockdown, as well as the prolonged isolation, may have had a disproportionate impact on this vulnerable group, putting them at a higher risk of behavioral problems exacerbations (33). DD is defined as having a significantly lower-than-average intellectual disability, and research shows that, when compared to children and adolescents with intellectual disabilities, children and adolescents with ASD have significantly higher rates of behavioral and emotional problems (34). ASD is more severe when compared to other disabilities. Individuals with ASD have differences in receptive communication skills and may experience delays in processing information (35), which affects their ability to respond to the pandemic in an accepting and efficient manner (8, 36). Our findings add to the growing body of evidence that people with severe disabilities, such as ASD, are disproportionately affected by negative events (37, 38).

Third, some families stopped recovering, but most of the families were undertaking the rehabilitation recovery (77.6% for ASD group vs. 72.86% for DD group). The implementation of an online form of education during the COVID-19 pandemic was less than optimal for children. The literature on the efficacy of telehealth for individuals with ASD is mixed (39, 40). The implementation of home education presents its challenges, even for the professional developmental behavioral pediatrician (8). Home education for children with special needs should be more focused on parental roles (41), as the online advice from others only decreases parents’ responsibilities. For children who rely on gestures or picture exchange, they often require a parent or additional support to help them to communicate responses. This study also collected parents’ narratives on their perceived needs throughout the pandemic through an open-ended question. About half of the participants reported needing one-to-one support and guidance from professionals online, where the answers were consistent with those of earlier studies that stated that healthcare services, especially in-home services, were badly needed (31); this also reflected that those parents were not satisfied with the current online support. Delivery of programs that are easily implemented and meet the needs of children and their families is needed (42). Although clinical trials and systematic reviews have shown telehealth interventions to result in promising improvements in learning under the circumstances of limited access to in-person services, we have not obtained optimistic results in the case of a real scenario (COVID-19). It also reminds us of the differences between the real world and experiments.

Fourth, we found that low family income increased the risk for negative changes in children’s emotions and behavior. This finding aligned with recent research highlighting significant socioeconomic health inequities in other populations (e.g., adults) during the pandemic (43, 44). Parents with low income who have children with ASD may be more vulnerable to employment loss and may experience greater challenges finding care for their child. This in turn can exacerbate behavioral challenges in these children, adding to the myriad of challenges already faced by caregivers (8, 45, 46).

Fifth, the increase in time spent with the child was conducive to the improvement of the emotional functions and skills of children with ASD during the pandemic. With long-term restrictions at home, parents had more time to spend with their children, thus more family activities could be done. The results of the British Millennium Cohort study found that in the early development of children, a series of activities with parents and children had a significant effect on children’s cognitive development (47). Long-term home life also increases the chances of home language input. Home language input is positively correlated with children’s cognitive ability and vocabulary diversity (48). This had a positive effect on the development of children with ASD. But for children with DD, we did not find the accompanying time as an influence. This finding showed that different factors play a role in different kinds of developmental disorders.

### Limitations

First, the sample is drawn from a network survey. All families were invited to participate in this study and completed an electronic questionnaire, which did not allow for control stratification in sampling. Although the sex ratio and family location distribution of this study were consistent with the main data of the Second National Survey of Disabled People (SNSDP), we should adopt more rigorous sampling methods to improve our research in the future. Second, the data’s accuracy should be considered. A considerable amount of information was in the form of parents self-reporting, which might deviate from the real situation. Our questionnaire did not assess whether worsening changes reached a level of clinical significance or represented normative variations over time. Third, more studies following the cohorts of children longitudinally should be conducted to understand the long-term impacts of the COVID-19 pandemic on the functioning of children with ASD and DD as well as on their families. Fourth, the impacts of specific comorbidities of the children and parental genders were not further discussed in this article. In the future, better classification of comorbidities and respondent bias caused by gender will aid in the development of more appropriate procedures.

## Conclusion

In conclusion, the current survey study indicates that the ongoing COVID-19 pandemic has resulted in a difficult period for the majority of families with preschoolers with ASD and DD, showing increased emotional and behavioral problems. During the COVID-19 pandemic, the implementation of online education was less than ideal for children and even teachers’ online instruction was not as effective as parental-oriented instruction. More professional one-to-one online support and psychiatric care should be made available in the future. Our findings could help shape future policies and interventions to reduce the negative effects of COVID-19 on young people with developmental disorders and their families. More importantly, we should better evaluate the impact of policies on people’s health, take timely measures to protect the interests of vulnerable groups, and reduce health inequities.

## Data availability statement

The raw data supporting the conclusions of this article will be made available by the authors, without undue reservation.

## Ethics statement

The studies involving human participants were reviewed and approved by the Ethics Committee of Peking University Institutional Review Board and approval number is IRB00001052-20016. Written informed consent to participate in this study was provided by the participants or their legal guardian/next of kin.

## Author contributions

YZ conceptualized and designed the study, drafted the initial manuscript, reviewed, and revised the manuscript. XZ and RZ designed the data collection instruments, collected the data, reviewed, and revised the manuscript. YL coordinated and supervised the data collection and critically reviewed the manuscript for important intellectual content. All authors approved the final manuscript as submitted and agreed to be accountable for all aspects of the work.
