# Supplementary material for: The impact of COVID-19 pandemic on emotional and behavioral problems of children with autism spectrum disorder and developmental delay aged 1–6 years in China
Source: Front Psychiatry. 2023 Feb 24;14:1134396. doi: 10.3389/fpsyt.2023.1134396 (PMC9998531; doi:10.3389/fpsyt.2023.1134396)
Supplement: Supplementary file 1 [file Table_1.DOCX]

**The impact of COVID-19 pandemic on emotional and behavioral problems of autism spectrum disorder and developmental delay children aged 1-6 years in China**

eTable 1. Distribution of sample by residence provinces

| Provinces | N | % | Region |
| --- | --- | --- | --- |
| Guangdong | 839 | 17.30 | Eastern |
| Shandong | 492 | 10.15 | Eastern |
| Anhui | 356 | 7.34 | Central |
| Hunan | 331 | 6.83 | Central |
| Jiangsu | 288 | 5.94 | Eastern |
| Henan | 259 | 5.34 | Central |
| Zhejiang | 249 | 5.14 | Eastern |
| Jiangxi | 247 | 5.09 | Central |
| Fujian | 192 | 3.96 | Eastern |
| Hubei | 179 | 3.69 | Central |
| Beijing | 162 | 3.34 | Eastern |
| Hebei | 161 | 3.32 | Eastern |
| Guangxi | 151 | 3.11 | Eastern |
| Sichuan | 132 | 2.72 | Western |
| Shanxi^a^ | 121 | 2.50 | Central |
| Shanxi^b^ | 109 | 2.25 | Western |
| Heilongjiang | 99 | 2.04 | Central |
| Chongqing | 75 | 1.55 | Western |
| Tianjin | 74 | 1.53 | Eastern |
| Hainan | 62 | 1.28 | Eastern |
| Liaoning | 48 | 0.99 | Eastern |
| Shanghai | 39 | 0.80 | Eastern |
| Jilin | 31 | 0.64 | Central |
| Guizhou | 31 | 0.64 | Western |
| Inner Mongolia | 29 | 0.60 | Central |
| Xinjiang | 27 | 0.56 | Western |
| Gansu | 24 | 0.49 | Western |
| Yunnan | 22 | 0.45 | Western |
| Ningxia | 18 | 0.37 | Western |
| Xizang | 1 | 0.02 | Western |
| Qinghai | 1 | 0.02 | Western |
| Total | 4849 | 100.00 |  |

Abbreviation: N: number.

a: In Chinese, it is “山西” Shanxi Province

b: In Chinese, it is “陕西” Shanxi Province

eTable 2. Data quality exploration

|  | **Study population** | | **The Second China National Sample Survey on Disability ^a^** | |
| --- | --- | --- | --- | --- |
|  | n | % | n | % |
| **District** |  |  |  |  |
| Eastern | 2,350 | 56.8 | 25,713^b^ | 62.3 |
| Central | 1,411 | 34.1 | 9,747^b^ | 23.6 |
| Western | 377 | 9.1 | 5,834^b^ | 14.1 |
| **Sex** |  |  |  |  |
| Boy | 3,451 | 83.4 | 32,130^b^ | 77.8 |
| Girl | 687 | 16.6 | 9,163^b^ | 22.2 |
| Sex-Ratio | 5.02 |  | 3.51^b^ |  |
| **total** | **4,138*** |  | **41,293^b^** |  |

Abbreviation: n: number.

a. The second national sample survey of the disabled began at 0:00 on April 1st, 2006 and ended on May 31st, 2006. The disabled sample was 252,6145. A total of **73** children aged 0-6 diagnosed with autism. All diagnoses were made by psychiatrists according to ICD-10.

b. weighted samples by Chinese population, which was not the actual number of screening people.

* only for ASD group
